# Dieting Practices of Adolescents Seeking Obesity Treatment

**DOI:** 10.3390/nu17193100

**Published:** 2025-09-29

**Authors:** Hiba Jebeile, Eve T. House, Louise A. Baur, Cathy Kwok, Clare E. Collins, Sarah P. Garnett, Natalie B. Lister

**Affiliations:** 1 Children’s Hospital Westmead Clinical School, The University of Sydney, Sydney, NSW 2145, Australia; eve.house@sydney.edu.au (E.T.H.); louise.baur@health.nsw.gov.au (L.A.B.); cathy.kwok@sydney.edu.au (C.K.); sarah.garnett@sydney.edu.au (S.P.G.); natalie.lister@sydney.edu.au (N.B.L.); 2 School of Health Sciences, College of Health, Medicine and Wellbeing, University of Newcastle, Newcastle, NSW 2308, Australia; clare.collins@newcastle.edu.au

**Keywords:** dieting, adolescent, obesity, disordered eating

## Abstract

**Background**: Adolescents with obesity participate in self-directed weight loss attempts, and these may be associated with disordered eating. This study aimed to understand prior engagement with a dietitian and previous dieting practices of adolescents presenting for obesity treatment. Additionally, we aimed to understand the association between prior dieting and eating disorder risk, binge eating, weight bias internalisation and body image. **Methods**: This cross-sectional study included 141 adolescents (median [IQR] age: 14.8 [13.8–15.7] years) with BMI 35.28 (31.99–38.57) kg/m^2^ and ≥1 related complication presenting for a prescriptive dietary intervention. Adolescents were asked whether they had previously seen a dietitian (yes/no) and/or previously trialled any other diets for weight management. Associations between reported diets and the Eating Disorder Examination Questionnaire (EDE-Q), the Binge Eating Scale, the Weight Bias Internalisation scale and Body Appreciation Scale scores were assessed using multiple one-way ANOVAs. **Results**: A total of 68 (48.2%) adolescents had previously seen a dietitian and 106 (75.2%) had trialled at least one diet. Most adolescents had used one diet type (*n* = 74; 52.5%), and 29 (20.6%) had used two or three different diets. Most adolescents reported following a healthy eating pattern (*n* = 76; 53.9%), with 11 trying a low-carbohydrate diet (7.8%) or a specific eating plan, e.g., low sugar, vegetarian (*n* = 11; 7.8%). There were no associations between dieting attempts and scores of measures of disordered eating, weight bias or body appreciation. **Conclusions**: Many adolescents presenting for obesity treatment will have trialled diets, with or without the support of a dietitian. Clinicians providing nutrition education and prescribing dietary interventions should be aware of this and the potential influence on adolescent perceptions of dieting practices.

## 1. Introduction

The prevalence of obesity is forecast to increase in Australia and globally over the next decade [1]. Many adolescents also experience weight-related comorbidities, including insulin resistance, metabolic-associated fatty liver disease and dyslipidaemia, warranting treatment [2]. First-line treatments include intensive behavioural interventions, delivered by a multidisciplinary team, and addressing diet, physical activity, sleep and behaviour change [3,4]. Prescribed dietary interventions, pharmacotherapy and bariatric surgery may be used for adolescents with moderate to severe obesity and related complications; however, access to these therapies in Australia remains limited [3,5,6].

With limited access to supportive medical care, prior research has explored the self-directed weight loss practices of adolescents. Analysis of data from 6117 children and adolescents (8–15 years) captured as part of the National Health and Nutrition Examination Survey (NHANES) data in the United States, found that half of children and adolescents had attempted weight loss previously, which increased to 75% of adolescents with obesity [7]. Weight loss attempts included exercising (93%), reducing sweets or fatty foods (82%) or reducing overall food intake (71%), and 45% reported following a specific diet and 35% reported skipping meals [7]. Similarly, from 5275 adolescents with obesity surveyed as part of the global ACTION Teens study, 58% reported a recent weight loss attempt, with 60% seeing a health care provider (primarily the adolescent’s doctor [33%] or a dietitian/nutritionist [23%]) about weight concerns [8]. As part of weight loss attempts, 17% had followed a diet program, 14% followed an exercise program, 14% had used weight loss medication (8% over the counter, 6% prescribed) and 2% had bariatric surgery [8]. However, these studies focus on dieting behaviours or dietary intake and do not capture the specific diet types or level of restriction used by adolescents. Understanding specific diet types and practices used by adolescents can inform diet-related education needs of adolescents presenting for obesity treatment.

When measured in community samples, reported use of dieting practices is associated with a higher risk of disordered eating, including binge eating [9,10], and dieting is an established risk factor for eating disorders [11]. Additionally, internalisation of negative societal attitudes and stigma towards higher weight is associated with disordered eating [12] and may lead to dieting attempts to lose weight. In contrast, positive body image is associated with lower levels of disordered eating and is linked to healthier lifestyle patterns [13]. Therefore, the aim of the current study was to understand prior engagement with a dietitian and report any previous dieting practices of adolescents presenting for obesity treatment. A secondary aim was to understand the association between prior dieting and eating disorder risk, binge eating, weight bias internalisation and body image.

## 2. Materials and Methods

Adolescents (13 to 17 years) with obesity and ≥1 metabolic complication were recruited into the Fast Track to Health randomised trial in Sydney and Melbourne, Australia, between 31st January 2018 and 31st March 2023. The trial protocol has been published, the study was approved by The Sydney Children’s Hospitals Network Human Research Ethics Committee (HREC/17/SCHN/164) and the primary outcome, a change in BMI z-score at 52 weeks, has been previously reported [14,15,16]. Agreement to participate was obtained from adolescents, and written consent was obtained from parents/carers.

Fast Track to Health was a 52-week intensive behavioural intervention comparing two dietary approaches, intermittent and continuous energy restriction. There were 141 adolescents recruited into the trial with a median (IQR) age of 14.8 (13.8–15.7) years, BMI 35.28 (31.99–38.57) kg/m^2^ and BMI z-score 2.30 (2.03–2.69) at baseline. At the time of recruitment, adolescents were not engaged in another weight loss attempt.

At baseline, as part of the initial consultation with the study dietitian, adolescents were asked two questions about prior engagement with a dietitian and dieting history: (1) if they had ever previously consulted with a dietitian (Yes/No), and if yes, in what context; and (2) an open ended question about whether they had previously trialled any specific diets, and if yes, clarifying questions were asked to determine the type of diet trialled. Participant descriptions of the diets trialled were recorded, coded and categorised into common diet types or into dietary patterns (Table 1). For example, ‘healthy eating’ was coded if adolescents reported trying to improve their diet quality and ‘eat healthier,’ while if they reported ‘calorie counting,’ this was coded as a reduced-calorie diet. The number of different diets trialled by each participant was also coded. At baseline, adolescents completed the Eating Disorder Examination Questionnaire (EDE-Q) [17,18,19], Binge Eating Scale [20,21,22], Weight Bias Internalisation Scale [23,24] and the Body Appreciation Scale [25,26], to assess disordered eating, weight bias internalisation and attitudes towards their body, respectively.

Statistical analyses were performed using IBM SPSS Statistics, V28.0 (Chicago, IL, USA). Descriptive statistics were used to summarise the proportion of adolescents who had previously seen a dietitian and trialled different diets or dietary patterns. To investigate the association between the number of diets trialled and age, BMI expressed as a percentage of the 95th percentile [27], disordered eating and body satisfaction outcomes, we conducted multiple one-way ANOVAs. Each ANOVA tested the differences between three groups: no diet, one diet and two–three diets. Assumptions of normality and homogeneity of variances were checked prior to analysis. Where assumptions were not met, non-parametric tests were used. Chi-squared test was used to test the association between sex and having seen a dietitian or attempting to diet. As the number of adolescents having trialled some diets was small (<5), Fisher’s exact test was used to test the association between the number of dieting attempts (one or two/three attempts) and the type of diet trialled. The Bonferroni correction was applied to account for multiple comparisons; an adjusted significance threshold of *p* < 0.01 was used. Data were available for all 141 adolescents on their history of consulting with a dietitian and for 138 adolescents on their dieting history.

## 3. Results

From 141 adolescents recruited into the Fast Track to Health Trial, participants had baseline median (IQR) scores on the EDE-Q of 2.28 (1.43–3.14), Binge Eating Scale of 11 (5–17; *n* = 135), Weight Bias Internalisation Scale of 3.91 (3.05–4.82; *n* = 137) and Body Appreciation Scale of 3.12 (2.65–3.79; *n* = 138). There were 68 (48.2%) adolescents who reported they had previously consulted with a dietitian in the community or private practice (*n* = 44, 31.2%) or in an obesity/weight management clinic (*n* = 23, 16.3%; Table 1). Most adolescents had trialled one type of diet (*n* = 73, 52.9%), with 30 (21.7%) reporting having used two or three different diets. There were 44 (31.9%) adolescents who had trialled at least one diet and had not consulted with a dietitian, and 27 (19.6%) had not seen a dietitian and had not used any diets.

There were no associations between sex (X^2^[1, *n* = 141] = 0.175, *p* = 0.68), age (seen a dietitian, *n* = 68 mean [SD] 179 [15] months; not seen a dietitian, 178 [15] months; F[df] 0.137 [1139], *p* = 0.712) or BMI, expressed as a percentage of the 95th percentile (seen a dietitian, *n* = 68, mean [SD] 132 [15]; not seen a dietitian, *n* = 73, 127 [15]; F[df] 4.303 [1139], *p* = 0.04) and history of consulting with a dietitian. There was no association between sex (X^2^[1, *n* = 138] = 3.820, *p* = 0.051) and attempting to diet, or between age and BMI, expressed as a percentage of the 95th percentile, and the number of diets used (Table 2).

### 3.1. Types of Diets

**Figure 1:**
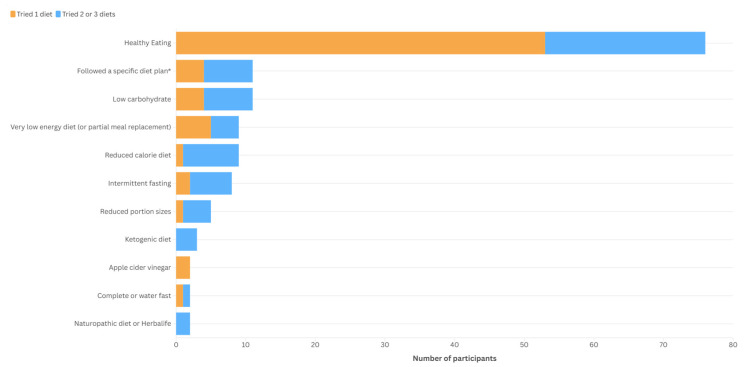
Types of diets used by adolescents prior to attending a weight management intervention grouped by the number of diets trialled (one diet or two/three diets). * Participants who have trialled multiple diets are represented more than once.

Most adolescents reported trying to follow a healthy eating pattern (*n* = 76, 55.1%), with fewer following specific dietary patterns (Table 1, Figure 1). Adolescents who had trialled two or three different diets were more likely to try a reduced-calorie diet (Fisher’s Exact Test, *p* < 0.001) or intermittent fasting (*p* = 0.007) compared to adolescents trialing one type of diet. There were no differences for use of healthy eating (X^2^[1, *n* = 103] = 0.182, *p* = 0.67), reduced portion sizes (*p* = 0.024), following a specific dieting plan (*p* = 0.013), low carbohydrate (*p* = 0.013) or VLED (*p* = 0.442) based on the number of diets trialled. Numbers within these groups are small and should be interpreted with caution (Figure 1).

### 3.2. Psychosocial Wellbeing and Eating Behaviours

**Figure 2:**
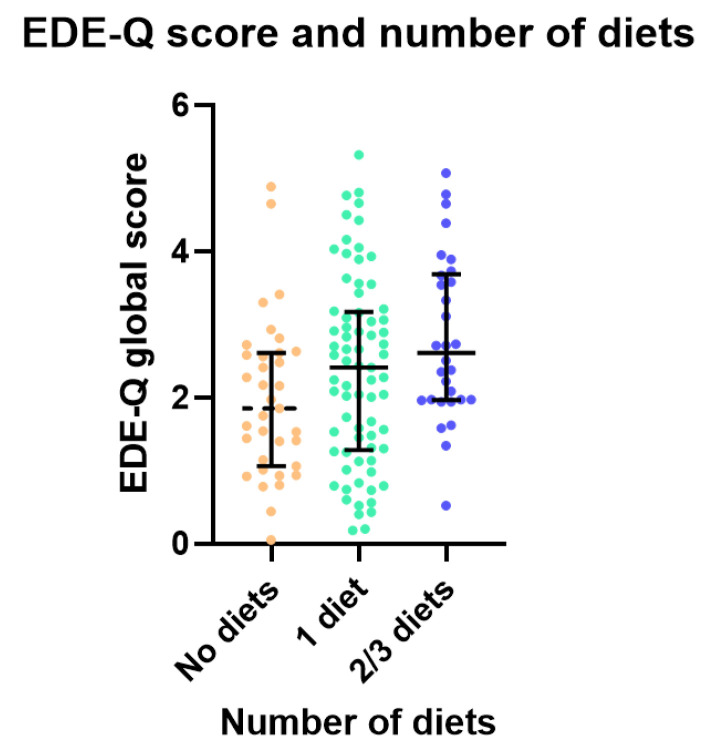
Eating Disorder Examination Questionnaire scores by number of diets trialled.

There were no differences in scores on the Eating Disorder Examination Questionnaire (Figure 2), Binge Eating Scale, Weight Bias Internalisation Scale and Body Appreciation Scale based on the number of diets trialled (*p* > 0.01, Table 2).

## 4. Discussion

This study aimed to explore dieting practices of adolescents presenting to specialist obesity treatment services within two tertiary hospitals in Australia. Half of adolescents had previously consulted with a dietitian and most had trialled one or more dietary interventions in an attempt to reduce body weight. It is promising that most adolescents had used a healthy eating plan, though some were engaging in restrictive dieting practices. There was no association between age, sex or BMI expressed as a percentage of the 95th percentile, disordered eating, weight bias internalisation or body appreciation and the number of diets trialled. The results of the current study highlight that adolescents were using weight loss diets, often multiple times, prior to engagement with a multidisciplinary weight management program. These previous dieting experiences, particularly for those who have not received dietetic support, should be addressed prior to obesity treatment to understand adolescent perceptions of weight management and nutrition-related and/or eating behaviours.

The proportion of Australian adolescents following specific diet plans (44%) is similar to that reported by adolescents from the US (45%) [7] and double those from the ACTION Teens study (17%) [8]. As adolescents were enrolled in a clinical trial testing two prescriptive dietary approaches, the adolescents recruited may be those who are more motivated to use diets. Additionally, recruited adolescents had obesity related complications; it is possible they had a longer duration of obesity and/or greater severity than those represented in prior studies. Although it is promising that healthy eating was the most common approach used, many adolescents had trialled restrictive diets, including low-carbohydrate approaches and very-low-energy diets, which may be associated with some side effects [28]. Some of these restrictive approaches appear more common among adolescents who have trialled two or three diets. These approaches are recommended in treatment guidelines for adolescents with severe obesity and/or obesity related complications, but only under the guidance of trained clinicians [5]. Clinicians providing nutrition education and prescribing diet interventions, in the context of obesity treatment, should be aware of these prior dieting attempts and the potential influence on adolescent perceptions of healthy and unhealthy dieting practices.

In community samples, the association between adolescent self-directed dieting attempts and BMI change is inconsistent. Some studies have found dieting was associated with increased BMI [10,29], and another found dieting was associated with a reduction in BMI z-score among adolescent girls [30]. Similarly, dieting has been associated with disordered eating in community samples [9,10]. Therefore, it is concerning that many adolescents had not sought professional support prior to presenting to a specialist obesity treatment service. This may be representative of the lack of paediatric obesity services in Australia [6]. Although in this study, we did not find an association between the number of dieting attempts and disordered eating, in contrast to prior observational data showing that dieting is associated with binge eating [9,10]. It is possible that this study was underpowered to detect this, or that adolescents presenting to obesity treatment are different from those recruited for eating disorder-focused studies. Future research should consider this in a larger sample, as well as whether specific diets have more or less of an influence on disordered eating and weight-related outcomes. As part of this study and previous research, data have not captured the age of onset of dieting, duration of the diet attempt, where information was sought or any support that was provided. Additionally, we did not capture whether adolescents achieved the desired weight loss, health improvement or other unintended outcomes with these dietary approaches. These are important factors to capture in future research to inform our understanding of dieting practices and how these may contribute to eating behaviours, disordered eating and psychosocial health. Sources of information may be particularly important to consider at the present time, as adolescents report accessing nutrition information via social media [31], yet this has been shown to lack helpful evidence-based information [32,33,34], and to contribute to disordered eating and poor body image [35,36]. To our knowledge, a standardised assessment is not available to capture these nuanced details on adolescent dieting behaviours. Further research is needed to understand how to collect these data, and the influence of these behaviours on dietary intake and eating behaviours.

This study used a structured protocol to understand adolescent dieting behaviours and the types of diets trialled within a sample of adolescents presenting to a clinical trial. There are several limitations. Analyses are exploratory and would ideally be replicated in a larger sample. Responses may be subject to recall bias as adolescents were asked to retrospectively report the type of diets trialled. We did not capture if consultation with a dietitian was linked to the diets trialled, the number of dieting attempts (i.e., if the same diet was used multiple times), frequency of dieting behaviour, the length of time the adolescent had been dieting prior to enrolment in the trial, or if they achieved weight loss or health improvement with the dietary approach used. Additionally, adolescents were recruited into a diet intervention study, possibly capturing adolescents with an interest in diet, which may have led to sampling bias. Standardised methods of capturing the history of dieting will improve understanding of adolescent dieting practices and data collection approaches.

## 5. Conclusions

For many adolescents presenting for obesity treatment, this was their first consultation with a dietitian, though most had trialled one or more weight management diets prior to engagement with this professionally supported program. Within this sample, prior dieting was not associated with disordered eating, weight bias internalisation or body appreciation. Standardised assessment approaches and further research are needed to better understand adolescent dieting practices, including frequency and duration of dieting attempts and sources of information. Clinicians providing obesity treatment should be aware of prior dieting attempts and address this as part of normalising healthy eating behaviours.

## Figures and Tables

**Table 1:** Dieting attempts of adolescents presenting for obesity treatment.

**Variable, *n* = 141**	***n*(%)**
**Consulted a dietitian—yes**	**68 (48.2%)**
- Obesity or weight management clinic	23 (16.3%)
- Community program or private practice	44 (31.2%)
- Other, non-weight-related	3 (2.1%)
Number of diets trialled by adolescents,* *n* = 138	***n*(%)**
0	35 (25.4%)
1	73 (52.9%)
2	26 (18.8%)
3	4 (2.9%)
Previous diets, *n* = 138 *	
None	35 (25.4%)
Healthy eating	76 (55.1%)
Low carbohydrate	11 (7.8%)
Followed a specific diet plan **	11 (8.0%)
Very-low-energy diet (or partial meal replacement)	9 (6.5%)
Reduced-calorie diet (with calorie target)	9 (6.5%)
Intermittent fasting	8 (5.8%)
Ketogenic diet	3 (2.2%)
Reduced portion sizes	5 (3.6%)
Naturopathic diet or Herbalife	2 (1.4%)
Apple cider vinegar	2 (1.4%)
Complete or water fast	2 (1.4%)

* Participants are represented more than once where more than one type of diet has been trialled; ** specific diet plans included use of meal replacements, low sugar diet, GM diet, psoriasis diet, vegetarian and medication.

**Table 2:** Mean scores on disordered eating and body image measures by dieting status (one-way ANOVA).

Variable, Mean (SD)	Non-Dieter, *n* = 35	One Diet, *n* = 73	Two–Three Diets, *n* = 30	F(df)	ANOVA, *p*-Value for Difference Between Groups
Age (months)	178 (14)	179 (16)	179 (14)	0.059 (2135)	0.943
BMI, as %95th percentile	128 (16)	131 (16)	129 (14)	0.617 (2135)	0.541
BES score *	10.16 (8.11)	11.48 (7.83)	13.09 (7.08)	1.153 (2129)	0.319
EDE-Q global score	1.98 (1.08)	2.36 (1.28)	2.81 (1.12)	3.871 (2135)	**0.023**
Weight bias internalisation score	3.86 (1.35)	3.95 (1.24)	4.15 (1.26)	0.462 (2131)	0.631
Body appreciation scale	3.15 (0.83)	3.21 (0.82)	3.11 (0.81)	0.176 (2132)	0.839

* BES was not normally distributed. A Kruskal–Wallis test revealed no statistically significant differences between groups, H(2) = 2.89, *p* = 0.236.

## Data Availability

Data may be made available following additional ethics approvals.

## Abbreviations

The following abbreviations are used in this manuscript:

BMI	Body Mass Index
VLED	Very-Low-Energy Diet
EDE-Q	Eating Disorder Examination Questionnaire
